# Sex-associated effect of *CETP *and *LPL *polymorphisms on postprandial lipids in familial hypercholesterolaemia

**DOI:** 10.1186/1476-511X-8-24

**Published:** 2009-06-26

**Authors:** Katherine K Anagnostopoulou, Genovefa D Kolovou, Peggy M Kostakou, Constantinos Mihas, Georgios Hatzigeorgiou, Christina Marvaki, Dimitrios Degiannis, Dimitri P Mikhailidis, Dennis V Cokkinos

**Affiliations:** 1 1st Cardiology Department, Onassis Cardiac Surgery Center Athens, Greece; 2 Molecular Immunology Laboratory, Onassis Cardiac Surgery Center, Athens, Greece; 3 Internal Medicine Department, General Hospital of Kimi, Kimi, Greece; 4 Department of Nursing, Technical School of Education, Athens, Greece; 5 Department of Clinical Biochemistry (Vascular Prevention Clinics), Royal Free Hospital campus, University College London, London, UK

## Abstract

**Background:**

This study assessed the gender-specific influence of the *cholesteryl ester transfer protein *(TaqIB, I405V) and *lipoprotein lipase *(S447X) polymorphisms on the response to an oral fat tolerance test in heterozygotes for familial hypercholesterolaemia.

**Methods:**

We selected and genotyped 80 men and postmenopausal women heterozygous for familial hypercholesterolaemia (main group) as well as 11 healthy control subjects. Patients were subgrouped based on their response to oral fat tolerance test. The oral fat tolerance test was defined as pathological when postprandial triglyceride concentration was higher than the highest triglyceride concentration observed in healthy subjects (220 mg/dl) at any time (2, 4, 6 or 8 h).

**Results:**

In the pathological subgroup, men had significantly higher incremental area under the curve after oral fat tolerance test than postmenopausal women. Furthermore, multivariate analysis revealed a gender association of TaqIB and I405V influence on postprandial lipaemia in this subgroup.

**Conclusion:**

In conclusion, it seems that gender and TaqIB polymorphism of the *cholesteryl ester transfer protein *gene were both associated with the distribution of triglyceride values after oral fat tolerance test, only in subjects with a pathological response to oral fat tolerance test. Specifically, men carrying the B2 allele of the TaqIB polymorphism showed a higher postprandial triglyceride peak and a delayed return to basal values compared with women carrying B2. However, further investigations in larger populations are required to replicate and confirm these findings.

## Background

The mechanisms that control the cholesteryl ester transfer protein (CETP) have attracted attention, since plasma CETP concentration is associated with increased risk for premature atherosclerosis [1]. The CETP concentration and activity in plasma is dependent on several factors such as environmental components including dietary cholesterol [2], alcohol [3], smoking and obesity [4], gender [5], and genetic influence (e.g. polymorphism of *CETP *gene) [6-8]. CETP plays a major role in the remodeling of lipoprotein particles by mediating the transfer of high density lipoprotein (HDL) cholesteryl esters. When the level of triglyceride (TG)-rich lipoproteins is normal, CETP transfers HDL cholesteryl esters preferentially towards LDL particles [9]. In contrast, when the levels of fasting [10-12] or postprandial [11,13] TG-rich lipoproteins are increased, CETP transfers HDL cholesteryl esters towards larger VLDL particles resulting in the formation of small dense LDL particles [9]. Indeed, in type IIB hyperlipidaemia during the fasting state, CETP is implicated in the intravascular formation of atherogenic, small dense LDL through an indirect mechanism involving an elevated rate of cholesteryl esters transfer from HDL to VLDL and more specifically, to large VLDL-1 particles [14]. Moreover, elevated levels of cholesteryl ester-enriched VLDL-1 are associated with enhanced formation of atherogenic, small dense LDL in hypertriglyceridaemic states and notably in hyperlipidaemias of phenotypes IIB and IV and in the dyslipidaemias of type 2 diabetes and metabolic syndrome [9]. As a consequence, therapeutic strategies have been developed to specifically influence the action of CETP [15].

*CETP *variants have a strong impact on CETP activity and thus on HDL cholesterol levels [7]. Several polymorphisms have been identified in the coding sequence of the *CETP *gene including I405V [16]. I405V has been associated with reduced CETP mass, increased HDL cholesterol levels and increased cardiovascular risk [6,17]. Another widely studied *CETP *polymorphism is TaqIB which may account for 5.8% of the variance in HDL cholesterol [8]. In normolipidaemic subjects, the absence of the TaqIB restriction site (B2 allele) is associated with decreased CETP activity, increased HDL cholesterol levels and reduced risk of coronary heart disease in males compared with B1 subjects [18].

Lipoprotein lipase (LPL) also plays a key role in lipoprotein metabolism since it hydrolyzes triglycerides from VLDL and chylomicrons and also removes lipoproteins from the circulation [19-21]. LPL influences the interaction between atherogenic lipoproteins and the cell surface as well as the receptors on the vascular wall, playing an important role in atherogenesis [22-24]. More than 60 different mutations of the *LPL *gene have been described to date and they can lead to a reduction in enzyme synthesis and activity. The S447X is one of the most frequent polymorphisms of *LPL *with an incidence of 17–22% in Caucasian populations [25,26]. However, its effect on LPL activity is controversial. When tested in vitro, this activity has been described both as increased [27,28] and decreased [29] but in the majority of studies it was unchanged [30,31] compared with the wild type protein. Some studies demonstrated that S447X polymorphism may be favorable to catabolism of VLDL, decreasing fasting concentrations of triglycerides [26,27,32,33] but it was not associated with the incidence of coronary artery disease [34]. The S allele has been associated with higher triglyceride levels [35], whereas the X allele has been related with anti-atherogenic lipid profiles and a modest reduction in coronary disease risk [36].

Patients who are heterozygotes for familial hypercholesterolaemia (hFH), and especially men, have more pronounced postprandial hypertriglyceridaemia than controls [37-40]. Additionally, men have higher fasting and postprandial TG levels compared with women, which was demonstrated in a previous study where hFH men were compared with age-matched premenopausal hFH women [41]. Furthermore, men develop atherosclerosis complications 10 to 15 years earlier than women either in the case of familial hypercholesterolaemia or not. The etiology for these differences could be attributed to gender specific responses to naturally occurring genetic polymorphisms involved in lipid metabolism. Thus, the natural genetic variation at the *CETP *and *LPL *loci could be used to understand their possible impact on disease. Such human genetic studies have demonstrated conflicting conclusions. The TaqI B2 allele has been previously associated with protection against an exaggerated postprandial TG rise and a subsequent lowering of the HDL cholesterol levels in patients with increased TG concentrations (fasting and/or postprandial), by our group [42]. This was attributed to the B2 lowering-effect on CETP mass and activity which may become more prominent when TG levels are beyond normal [42].

The aim of the current study was to assess the sex-associated effect of the TaqIB as well as of two additional polymorphisms, the I405V of the *CETP *gene and the S447X of the *LPL *gene, on TG response to fat loading, in clinically defined hFH. A gender interaction was found with the TaqIB polymorphism of the *CETP *gene in patients with a pathological postprandial triglyceride response.

## Methods

### Subjects

Eighty hFH unrelated Greek Caucasian subjects (41 men and 39 postmenopausal women) as well as 11 healthy control subjects who underwent an oral fat tolerance test (OFTT) were genotyped. Some patients were previously examined for the impact of the *CETP *TaqIB polymorphism on postprandial plasma lipoprotein levels [42]. In the present study, the patients were further examined for 2 polymorphisms (*CETP *I405V and *LPL *S447X), including *CETP *TaqIB, however focusing on the gender-specific influence of these polymorphisms on postprandial lipaemia. Eleven healthy subjects (n = 6 men and n = 5 postmenopausal women) were also recruited and underwent an OFTT. Controls were normolipidaemic, free of any disease and had normal maximal treadmill exercise test. All patients were from the Lipid Clinic of Onassis Cardiac Surgery Center, Athens, Greece. The center's Institutional Review Board (IRB) approved the study and subjects gave their consent for the examination of their DNA. The diagnosis of hFH was based on the following clinical criteria: a) fasting total cholesterol > 290 mg/dl and fasting LDL cholesterol > 190 mg/dl, b) presence of tendon xanthomas in the patient or in a 1st or 2nd degree relative, and, c) history of premature vascular disease in a 1st degree relative > 60 years or in 2nd degree relative > 50 years old [43]. Heavy alcohol drinking, liver and renal disease, obesity, diabetes mellitus, hypertension, hypothyroidism and professional sport activity were all criteria of exclusion. None of the patients were on hypolipidaemic treatment.

### OFTT protocol

All patients were examined in the outpatient clinic after a 12 h overnight fast. The fatty meal was consumed within 20 min and plasma TG concentrations were measured before and at 2, 4, 6 and 8 h after the fat load. During this 8 h period, the participants were not allowed to eat or smoke. They were only allowed to drink water. The content of the meal has been described elsewhere [44]. Briefly, the fatty meal was a slight modification of that introduced by Patsch et al. [45], consisting of 83.5% fat, 14.0% carbohydrates and 2.5% proteins and was given in a dose based on the patient's body surface area (350 g for 2 m^2^).

There are no official guidelines for the normal ranges of the OFTT. Therefore, postprandial TG levels were characterised pathological taking into account the current knowledge from up to date studies. Our OFTT studies on healthy subjects revealed a limit range of 220 mg/dl [44]. Additionally, several studies of others (reviewed in ref [46]) have also reported similar results. Consequently, TG response to OFTT was considered pathological, when any of the postprandial TG concentration (at 2, 4, 6 or 8 h) was higher than the highest TG concentration (220 mg/dl). All hFH patients were grouped according to gender (main group) and according to the OFTT response (2 subgroups).

### Determination of blood lipids and glucose

Plasma total cholesterol (TC), TG and HDL cholesterol were measured using enzymatic colorimetric methods on a Roche Integra Biochemical analyzer, with commercially available kits (Roche Diagnostics Gmbh, Mannheim, Germany). The serum LDL cholesterol levels were calculated using the Friedewald formula in patients with TG levels < 400 mg/dl. Apolipoprotein AI, B and lipoprotein (a) were measured by nephelometry (Nephelometer BN-100, Behring, Germany). Blood glucose was measured using the hexokinase method, with a Dade Behring reagent on a Dimension (Dade Behring, Liederbach, Germany) instrument. All samples were analyzed within 24 h. The OFTT protocol has been described elsewhere [44].

### Genotyping

The *CETP *(TaqIB, I405V) and *LPL *(S447X) polymorphisms were detected by using Polymerase Chain Reaction and Restricted Fragment Length Polymorphism analysis as previously described [3,7,47].

### Statistical Analysis

*Ad hoc *power analysis showed that in order to detect two-sided differences higher than 20% in the main outcome variable (TG-AUC) between the two main study groups (men and women), achieving statistical power equal to 85% at a significance level less than 0.05, we had to recruit at least 32 individuals for each group. Due to unexpected conditions, more people entered the study, potentially increasing its statistical power. Values of numerical characteristics were tested for normality by using the Shapiro-Wilk test. All variables deviated from normality, thus non-parametric statistics were used. The Mann Whitney U test was used for the comparison of numerical values between 2 groups and the Pearson's chi-square test was used for evaluating any association between categorical variables. Multiple logistic regression analysis was used in order to compare various characteristics between cases and controls, adjusting for the effect of age. All continuous variables are presented as medians and interquartile ranges (75^th ^– 25^th ^percentile), while categorical variables are presented as relative frequencies (percentages). Areas under the curve (AUC) for serial measurements of TG levels at baseline and after the fatty meal were calculated using the trapezoid rule. In order to assess the role of alleles on the TG levels, we performed multiple median (least absolute value) regression analysis for each study group (Pathological and normal response to OFTT) adjusting for age, gender and body mass index (BMI), where the TG-AUC was the dependent variable and the aforementioned parameters were independent variables, since they did not distribute normally. First and second order interactions between main effect explanatory variables (response to OFTT, gender and TaqIB allele) were also tested in a suitable multivariate model. The t statistic was calculated in order to assess the significance of each dependent variable in the models. The level of significance was *a priori *set at p < 0.05. No adjustment for multiple testing was applied (see Discussion). Data were analyzed using STATA™ (Version 9.0, Stata Corporation, College Station, TX 77845, USA).

## Results

### Study groups

#### Main group

This group consisted of 41 hFH men and 39 hFH women who underwent an OFTT. All women were postmenopausal in order to exclude hormonal influence on postprandial lipaemia. Women were defined as postmenopausal when they reported their last menses to have been at least 12 months earlier. They were not on hormone replacement therapy. However, hormone levels were not assessed to confirm reproductive status.

#### Subgroups

Twenty four out of 41 hFH men [hFH-Pathological (hFH-P) men] and 21 out of 39 hFH women (hFH-P women) had a pathological response to the OFTT (subgroup hFH-P). The remaining 17 hFH men [hFH-Normal (hFH-N) men] and 18 hFH women (hFH-N women) had a normal response to the OFTT (subgroup hFH-N).

#### Controls

This group consisted of 11 healthy subjects with a normal response to the OFTT.

All participants ingested the individually calculated fatty meal and tolerated it well.

### [1] Baseline clinical, biochemical characteristics and postprandial TG levels in hFH-P and hFH-N subgroups

The size of hFH-P and hFH-N groups was 45 and 46 subjects, respectively. The baseline clinical, biochemical characteristics and postprandial TG levels are shown in Table 1. Those participants with a pathological OFTT response had significantly larger waist perimeter, higher TG levels in all phases of the study, higher HDL, apolipoprotein B and glucose baseline levels, while they had significantly lower apolipoprotein A levels.

**Table 1:** Baseline clinical characteristics of the main group (pathological and normal OFTT response)

	Pathological n = 45	Normal n = 46	*p*
Age (years)	44.7(23.0)	46.6(24.5)	0.59
BMI (kg/m^2^)	25.4(4.3)	24.4(3.3)	0.06
Waist (cm)	90.9(14.0)	84.3(17.7)	0.01
TC (mg/dl)	320.3(55.5)	297.2(118.5)	0.10
TG0 (mg/dl)	155.8(87.0)	78.5(31.3)	< 0.01
TG2 (mg/dl)	257.3(128.0)	119(43.0)	< 0.01
TG4 (mg/dl)	310.9(120.0)	138.8(70.8)	< 0.01
TG6 (mg/dl)	291.4(149.0)	130.6(61.3)	< 0.01
TG8 (mg/dl)	221.1(147.5)	106.1(54.2)	< 0.01
TG AUC (mg/dl/h)	2080.9(853.0)	944.8(342.4)	< 0.01
TG i-AUC (mg/dl/h)	846.7(420.1)	351.4(142.4)	< 0.01
HDL (mg/dl)	44.2(17.5)	55.7(21.3)	< 0.01
LDL (mg/dl)	240.9(51.5)	225.2(111.7)	0.19
Apo A (mg/dl)	141.3(36.3)	156.5(37.4)	0.05
Apo B (mg/dl)	169.3(32.0)	147.8(56.9)	< 0.01
Lp (a) (mg/dl)	24.6(19.0)	28.6(27.1)	0.48
Glu (mg/dl)	92.4(15.5)	84.6(13.0)	0.01

Data are presented as medians (interquartile ranges). BMI: body mass index, TC: total cholesterol, TG: triglycerides, AUC: area under the curve, i-AUC: incremental AUC, HDL: high density lipoprotein cholesterol, LDL: low density lipoprotein cholesterol, Apo A: apolipoprotein A, Apo B: apolipoprotein B, Lp (a): lipoprotein (a), Glu: glucose, hFH: heterozygotes for familial hypercholesterolaemia.

### [2] Baseline clinical, biochemical characteristics and postprandial TG levels in main and both subgroups

The clinical and biochemical characteristics of the main group and the subgroups hFH-P and hFH-N are shown in Tables 1, 2, 3, 4. In the main group and subgroup hFH-P, women were approximately 21 years older and in subgroup hFH-N, they were 23 years older than men, as expected by the study design. In the main group and subgroup hFH-P, women had higher HDL cholesterol and apolipoprotein AI levels compared with men. In the subgroup hFH-N, women had lower BMI and higher HDL cholesterol levels than men. In the main group and both subgroups, men had higher waist circumference compared with women, though results were within normal range.

**Table 2:** Clinical characteristics of the main group (separated in men and women), total cases and controls

	Men n = 41	Women n = 39	*P*	Total cases n = 80	Controls n = 11	p*
Age (years)	33.5(11.5)	55.0(11.0)	< 0.01	43.0(24.0)	54.0(31.0)	0.03
BMI (kg/m^2^)	24.7(3.5)	24.3(4.5)	0.25	24.4(4.0)	25.6(2.0)	0.09
Waist (cm)	93.0(14.0)	78.5(13.0)	< 0.01	85.0(17.0)	93.0(26.5)	0.26
TC (mg/dl)	292.0(70.5)	319.0(45.0)	0.01	313.5(56.3)	215.0(39.0)	< 0.01
TG0 (mg/dl)	110.0(90.5)	99.0(83.0)	0.53	105.5(85.0)	68.0(33.0)	< 0.01
TG2 (mg/dl)	206.0(129.3)	158.0(131.8)	0.20	174.0(119.0)	108.0(53.8)	< 0.01
TG4 (mg/dl)	245.0(162.5)	195.0(142.5)	0.12	220.0(160.5)	134.0(52.0)	< 0.01
TG6 (mg/dl)	203.0(166.5)	165.0(136.0)	0.40	173.0(171.0)	140.0(63.0)	0.01
TG8 (mg/dl)	157.0(105.0)	134.0(111.0)	0.77	139.5(107.0)	87.0(68.0)	0.02
TG AUC (mg/dl/h)	1568.1(1225.7)	1360.6(809.0)	0.16	1407.9(995.5)	920.8(436.0)	< 0.01
TG i-AUC (mg/dl/h)	604.6(630.8)	521.9(420.2)	0.08	564.5(483.8)	346.5(131.7)	0.03
HDL (mg/dl)	40.0(14.5)	55.0(23.0)	< 0.01	48.0(18.5)	47.0(12.0)	0.26
LDL (mg/dl)	231.0(61.0)	238.0(45.0)	0.17	232.0(58.8)	135.0(29.0)	< 0.01
Apo AI (mg/dl)	132.0(29.3)	158.4(32.0)	< 0.01	145.5(40.7)	144.0(31.8)	0.22
Apo B (mg/dl)	153.0(32.5)	162.5(34.1)	0.27	156.9(35.0)	101.0(19.5)	< 0.01
Lp (a) (mg/dl)	16.0(15.1)	15.9(30.5)	0.23	16.0(20.3)	21.5(31.4)	0.69
Glu (mg/dl)	86.0(14.3)	90.0(17.0)	0.27	87.5(15.0)	81.5(12.8)	0.03
TaqIB allele frequency n (%)						
B1	43 (52.4)	45 (57.7)	0.50	88 (55.0)	7 (35.0)	0.08
B2	39 (47.6)	33 (42.3)		72 (45.0)	13 (65.0)	
I405V allele frequency n (%)						
I	54 (65.9)	50 (64.1)	0.82	104 (65.0)	16 (72.7)	0.43
V	28 (34.1)	28 (35.9)		56 (35.0)	6 (27.3)	
S447X allele frequency n (%)						
S	75 (91.5)	69 (88.5)	0.53	144 (90.0)	18 (81.8)	0.26
X	7 (8.5)	9 (11.5)		16 (10.0)	4 (18.2)	

Data are presented as medians (interquartile ranges). BMI: body mass index, TC: total cholesterol, TG: triglycerides, AUC: area under the curve, i-AUC: incremental AUC, HDL: high density lipoprotein cholesterol, LDL: low density lipoprotein cholesterol, Apo A: apolipoprotein A, Apo B: apolipoprotein B, Lp (a): lipoprotein (a), Glu: glucose, hFH: heterozygotes for familial hypercholesterolaemia.

*: All comparisons between cases and controls are age-adjusted.

**Table 3:** Clinical and metabolic characteristics of the hFH-P men and women (subgroup hFH-P).

	hFH-P men n = 24	hFH-P women n = 21	*p*
Age (years)	34.0(9.8)	55.0(11.5)	< 0.01
BMI (kg/m^2^)	25.4(4.5)	25.9(4.4)	0.34
Waist (cm)	98.0(13.5)	85.0(15.0)	0.01
TC (mg/dl)	295.0(73.5)	317.0(39.0)	0.07
TG0 (mg/dl)	138.5(89.5)	156.0(78.0)	0.66
TG2 (mg/dl)	244.5(116.0)	240.0(116.0)	0.54
TG4 (mg/dl)	308.5(130.8)	266.0(122.0)	0.12
TG6 (mg/dl)	288.0(95.0)	247.0(208.5)	0.38
TG8 (mg/dl)	202.0(87.0)	206.0(190.0)	0.86
TG AUC (mg/dl/h)	2010.2(635.3)	1665.7(958.6)	0.14
TG i-AUC (mg/dl/h)	934.2(427.1)	707.5(358.1)	0.04
HDL (mg/dl)	37.0(6.8)	55.0(20.0)	< 0.01
LDL (mg/dl)	211.5(61.8)	232.0(31.0)	0.23
Apo AI (mg/dl)	127.5(23.1)	162.5(27.1)	< 0.01
Apo B (mg/dl)	159.0(31.3)	165.0(47.9)	0.52
Lp (a) (mg/dl)	15.5(15.6)	15.9(30.5)	0.30
Glu (mg/dl)	90.0(10.0)	96.0(21.0)	0.26
TaqIB allele frequency, n (%)			
B1	26 (54.2)	25 (59.5)	0.61
B2	22 (45.8)	17 (40.5)	
I405V allele frequency, n (%)			
I	33 (68.8)	28 (66.7)	0.83
V	15 (31.2)	14 (33.3)	
S447X allele frequency, n (%)			
S	44 (91.7)	38 (90.5)	0.84
X	4 (8.3)	4 (9.5)	

Data are presented as medians (interquartile ranges). BMI: body mass index, TC: total cholesterol, TG: triglycerides, AUC: area under the curve, i-AUC: incremental AUC, HDL: high density lipoprotein cholesterol, LDL: low density lipoprotein cholesterol, Apo AI: apolipoprotein AI, Apo B: apolipoprotein B, Lp (a): lipoprotein (a), Glu: glucose.

**Table 4:** Clinical and metabolic characteristics of the hFH-N men and women (subgroup hFH-N).

	hFH-N men n = 17	hFH-N women n = 18	*p*
Age (years)	33.0(13.5)	55.5(13.8)	< 0.01
BMI (kg/m^2^)	24.5(2.9)	22.8(4.1)	< 0.01
Waist (cm)	91.0(13.0)	76.0(13.0)	< 0.01
TC (mg/dl)	292.0(64.0)	331.0(59.3)	0.08
TG0 (mg/dl)	76.0(30.5)	76.0(38.8)	0.91
TG2 (mg/dl)	128.5(24.8)	112.0(54.5)	0.25
TG4 (mg/dl)	145.5(63.5)	132.0(78.5)	0.21
TG6 (mg/dl)	131.0(60.5)	126.0(64.3)	0.96
TG8 (mg/dl)	103.0(57.5)	99.5(66.8)	0.46
TG AUC (mg/dl/h)	990.3(335.0)	874.0(351.6)	0.37
TG i-AUC (mg/dl/h)	377.3(161.5)	312.8(235.8)	0.17
HDL (mg/dl)	48.0(18.5)	60.0(24.0)	0.02
LDL (mg/dl)	232.0(66.5)	254.5(58.0)	0.26
Apo AI (mg/dl)	137.5(41.7)	158.4(45.8)	0.09
Apo B (mg/dl)	146.8(41.0)	158.5(40.1)	0.26
Lp (a) (mg/dl)	16.0(18.5)	18.4(28.8)	0.65
Glu (mg/dl)	82.0(11.5)	89.0(12.5)	0.35
TaqIB allele frequency, n (%)			
B1	17 (50.0)	20 (55.6)	0.64
B2	17 (50.0)	16 (44.4)	
I405V allele frequency, n (%)			
I	21 (61.8)	22 (61.1)	0.96
V	13 (38.2)	14 (38.9)	
S447X allele frequency, n (%)			
S	31 (91.2)	31 (86.1)	0.51
X	3 (8.8)	5 (13.9)	

Data are presented as medians (interquartile ranges). BMI: body mass index, TC: total cholesterol, TG: triglycerides, AUC: area under the curve, i-AUC: incremental AUC, HDL: high density lipoprotein cholesterol, LDL: low density lipoprotein cholesterol, Apo A: apolipoprotein AI, Apo B: apolipoprotein B, Lp (a): lipoprotein (a), Glu: glucose.

Concerning the OFTT response, no differences were found between men and women in the main group and in subgroup hFH-N (Tables 2 and 4, respectively). However, in the subgroup hFH-P, men had significantly higher TG-iAUC compared with women (Table 3).

### [3] Baseline clinical, biochemical characteristics and postprandial TG levels in main group and controls

The clinical and biochemical characteristics of the main group (total cases) and the controls are presented in Table 2. Patients were about 11 years younger and had significantly higher TC, TG, LDL and apolipoprotein B levels than controls. Patients had significantly higher postprandial TG levels at all hours, higher TG-AUC and TG-iAUC than controls (Table 2), as expected.

### [3] Allelic associations in main group and in both subgroups at baseline and postprandially

Patients were not divided based on genotype due to the small sample size. Regarding the allele frequencies of B1, B2 (*CETP*, TaqIB), I, V (*CETP*, I405V) and S, X (*LPL*, S447X), there were no gender differences in the main group and both subgroups (Tables 2, 3, 4). It should be also reported that all allele frequencies in hFH with normal response did not differ significantly compared with those in controls.

**Figure 1:**
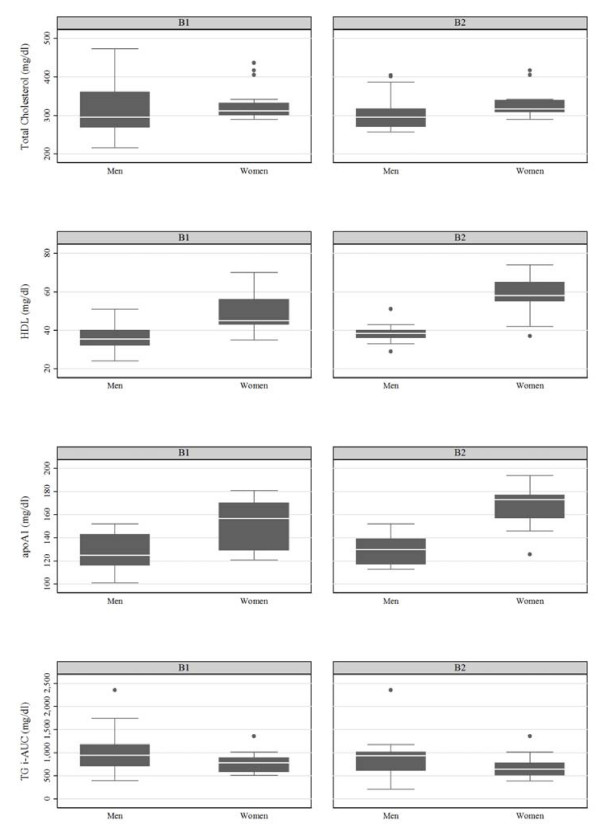
**Lipid values in hFH-P men and women with the B1 or B2 alleles**. Number of B1 and B2 alleles in men are 26 and 22, and in women are 25 and 17, respectively.

**Figure 2:**
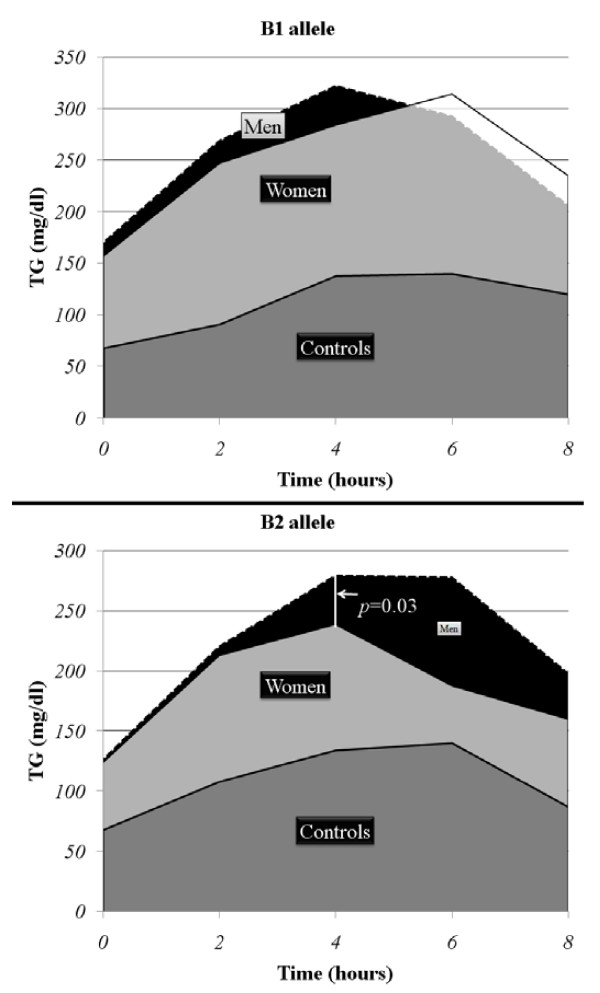
**Gender differences in the response of OFTT in hFH-P patients with B1 or B2 alleles**. TG: triglyceride, TGL: triglyceride, HDL: high-density lipoprotein cholesterol, apo AI: apolipoprotein AI.

**Figure 3:**
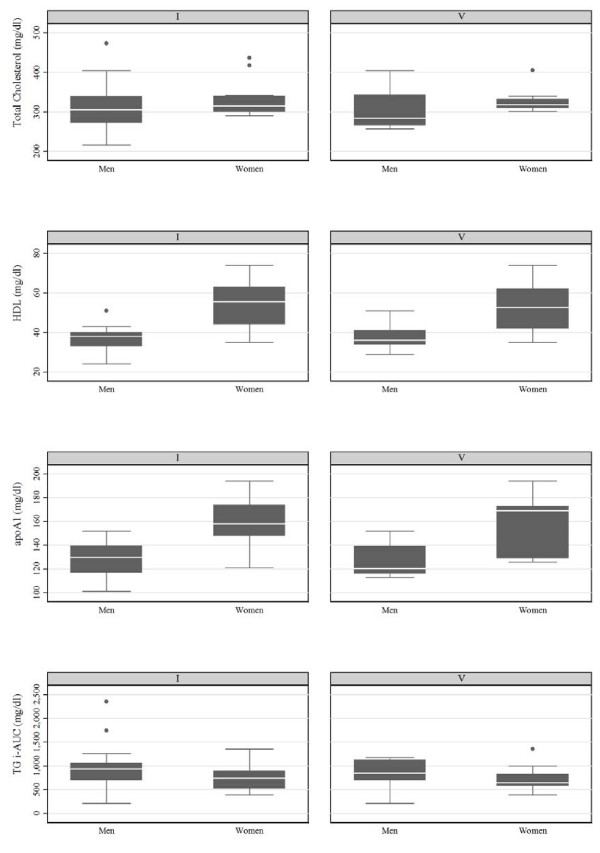
**Lipid values in hFH-P men and women with the I or V alleles**. Number of I and V alleles in men are 33 and 15, and in women are 28 and 14, respectively.

There wasn't any association between with B1, B2 (TaqIB), I, V (I405V) and S, X (S447X) carriers and OFTT response influenced by gender in the main group. Concerning the subgroup hFH-P, women carrying the B2 (TaqIB) allele had higher TC levels (317 ± 36 vs. 295 ± 53 mg/dl, p = 0.05) lower TG levels at the 4^th ^hour (239 ± 65 vs. 279 ± 95 mg/dl, p = 0.03) after fat load, compared with men with the B2 allele (fig 1 and fig 2). No other differences between men and women were observed. Women carrying either the B1 or B2 allele had higher HDL cholesterol (45 ± 14 vs. 36 ± 10 mg/dl, p < 0.01, 58 ± 12 vs. 39 ± 5 mg/dl, p < 0.01) and higher apolipoprotein AI levels (157 ± 41 vs. 125 ± 27 mg/dl, p < 0.01, 173 ± 27 vs. 130 ± 22 mg/dl, p < 0.01) compared with men carrying either the B1 or B2 allele, respectively (fig 1). Women with the I (I405V) allele had lower TC levels (315 ± 40 vs. 305 ± 69 mg/dl, p = 0.04) compared with men carrying the I allele (fig 3). Regarding the rest of lipid parameters, women carrying either the I or V allele had higher HDL cholesterol (56 ± 20 vs. 38 ± 8 mg/dl, p < 0.01, 53 ± 22 vs. 36 ± 7 mg/dl, p = 0.01), higher apolipoprotein AI (158 ± 26 vs. 130 ± 23 mg/dl, p < 0.01, 169 ± 47 vs. 120 ± 23 mg/dl, p = 0.01), and lower incremental TG-AUC (743 ± 363 vs. 934 ± 361 mg/dl, p = 0.04, 646 ± 285 vs. 844 ± 427 mg/dl, p = 0.05), compared with men carrying either the I or V allele, respectively (fig 3). No association of the S447X polymorphism with gender and OFTT was found.

Concerning the subgroup hFH-N, women carrying either the B1 or B2 allele had higher HDL cholesterol levels, compared with men carrying either the B1 or B2 allele (54 ± 23 mg/dl vs. 46 ± 17 mg/dl, p = 0.02, 70 ± 26 mg/dl vs. 50 ± 20 mg/dl, p = 0.01, respectively). No association of the TaqIB polymorphism with gender and OFTT was found. Regarding the I405V polymorphism, no difference was found between genders in I carriers. Compared with men carrying the V allele, women with the V allele had higher HDL cholesterol levels (70 ± 22 mg/dl vs. 46 ± 14 mg/dl, p < 0.01). No association of the I405V and S447X polymorphisms with gender and OFTT was found.

### [4] Multivariate analysis

In the total sample, TG response, gender and TaqIB allele had significant effect on TG – AUC (main effects) (coefficients = -678.6, 403.4, 678.1, p < 0.01, 0.01, < 0.01 respectively) (Table 5). In addition, the interaction between TG response and TaqIB allele was also significantly associated with the dependent variable. This indicates that the difference in TG-AUC levels between participants with normal and pathological TG response depends on their allele, since their interaction term is significantly associated with the dependent variable.

**Table 5:** Median (least absolute value) multiple regression by total sample based on the response to OFTT.

**Explanatory variables**	**Category**	**Coefficient**	**Standard Error**	***P***	**[95% Confidence Interval]**	
Response to OFTT	Pathological vs. normal	-678.6	161.8	< 0.01	-998.1	-359.1
Gender	Men vs. women	403.4	156.9	0.01	93.6	713.1
TaqIB allele	B1 vs B2	678.1	158.3	< 0.01	365.6	990.7
Response to OFTT × gender	Pathological & men vs. rest	-345.4	218.5	0.12	-776.8	86.1
Response to OFTT × TaqIB allele	Pathological & B1 vs. rest	-678.1	221.9	< 0.01	-1116.3	-240.0
TaqIB allele × gender	B1 & men vs. rest	-344.6	213.8	0.11	-766.9	77.8
Response to OFTT × TaqIB allele × gender	Pathological & B1 & men vs. rest	378.5	302.4	0.21	-218.8	975.7
Constant		1552.5	118.7	< 0.01	1318.1	1786.9

Triglyceride under the curve (TG-AUC) is the dependent variable.

OFTT: oral fat tolerance test, TG: triglyceride, AUC: area under the curve, BMI: body mass index

In the subgroup hFH-P, the presence of the B2 allele was significantly related to lower levels of TG-AUC (p = 0.01), adjusting for age (insignificant effect, p = 0.38), gender (females had significantly lower levels of TG-AUC, p = 0.05) and BMI (slight impact, p = 0.14) (Table 6).

**Table 6:** Median (least absolute value) multiple regression by group with pathological response to OFTT (subgroup hFH-P) based on the response to OFTT.

**Explanatory variables**	**Coefficient**	**Standard Error**	***p***	**[95% Confidence Interval]**	
B2 vs B1	-473.7	175.6	0.01	-824.0	-123.5
V vs I	-4.8	181.6	0.98	-367.0	357.3
Women vs Men	-633.9	313.4	0.05	-1259.0	-8.8
BMI	54.0	36.2	0.14	-18.1	126.1
Age	10.3	11.7	0.38	-13.1	33.6
Constant	1855.8	934.8	0.05	-8.5	3720.1

Triglyceride under the curve (TG-AUC) is the dependent variable.

In the subgroup hFH-N, although age and female gender had a significant relationship with TG-AUC (coefficient = 11.68, -366.25, p = 0.01, p = 0.03, respectively), the B1/B2 allele was not significantly associated with TG-AUC levels in this group (p = 0.55). BMI did not predict TG-AUC (p = 0.46) (Table 7). No association of the I405V polymorphism with TG-AUC was found in both models.

**Table 7:** Median (least absolute value) multiple regression by group with normal response to OFTT (subgroup hFH-N) based on the response to OFTT.

**Explanatory variables**	**Coefficient**	**Standard Error**	***p***	**[95% Confidence Interval]**	
B2 vs B1	-64.7	107.3	0.55	-279.6	150.2
V vs I	135.4	120.0	0.26	-105.1	375.8
Women vs Men	-366.3	160.2	0.03	-687.1	-45.4
BMI	-20.8	28.2	0.46	-77.2	35.6
Age	11.7	4.5	0.01	2.8	20.6
Constant	1399.8	734.8	0.06	-72.1	2871.7

Triglyceride under the curve (TG-AUC) is the dependent variable.

## Discussion

We assessed the gender-specific influence of TaqIB and I405V polymorphisms of the *CETP *gene and S447X polymorphism of the *LPL *gene, on postprandial TG levels in hFH subjects. To decrease the putative sex hormone influence, men were compared with postmenopausal women. We found that postmenopausal women seem to lose their sex-related advantage concerning the OFTT and have similar TG response compared with men. Furthermore, in subjects with a pathological response to OFTT, men carrying the B2 allele of the TaqIB polymorphism showed a higher postprandial TG peak and a delayed return to basal values compared with women carrying the B2 allele. In contrast, there was no gender association between postprandial TG levels and the S447X polymorphism of the *LPL *gene.

Previous studies concerning hFH subjects and postprandial lipaemia, showed a gender-specific influence on OFFT [39,40]. Postmenopausal hFH women and hFH men had a similar TG response to OFTT. Many studies [48-50], including ours [12,51], have shown that the magnitude of postprandial TG depends on fasting TG levels. Ageing in women leads to a larger variation in fasting TG levels, suggesting that with the loss of endogenous estrogens, the tight regulation of plasma TG may be lost [52]. Stevenson et al. [53] found a 12% increase of fasting TG levels after menopause, and others have reported even higher values [52,53]. Consequently, it seems that the menopause is associated with reduced protection against postprandial lipaemia. In the present study, hFH women and hFH men had similar fasting TG levels (118 ± 55 mg/dl vs. 125 ± 63 mg/dl, respectively, p = NS), which suggests that these TGs are already increased, since hFH premenopausal women tend to have lower fasting TG concentrations [53]. Furthermore, there was no gender difference in allele frequency of both polymorphisms of the *CETP *gene in any group. Since both polymorphisms of the *CETP *gene were not sex-related, the lack of any association with gender was expected. The novel finding of this study is that a significant gender association was found in TG response to OFTT in hFH-P subjects and the TaqIB polymorphism of the *CETP *gene. This suggests that the modulation of *CETP *polymorphisms on pathological postprandial TG levels depends on gender, documented also by median regression analysis (p < 0.001). This gender difference is probably not mainly related to sex hormones, since the women were postmenopausal. There are probably other mechanisms beyond sex hormones but still gender-dependent which can influence TG postprandially. For example, there could be different levels or localization of *CETP *expression. The main expression of *CETP *takes place in adipose tissue, where the promotion of selective uptake of HDL occurs [54,55]. Small, lipid poor adipocytes, in abdominal fat, express higher levels of *CETP *messenger RNA and may play major role in men [56]. Several studies found that higher postprandial TG levels associate with higher activity and concentration of CETP [57-60]. Others reported a positive correlation between the percentage of meal fatty acids and visceral fat mass [53,61]. However, they could not distinguish whether there are differences between men and women in fat uptake from subcutaneous fat. They also observed that women have more increased adipose tissue blood flow in the postprandial period than men. A greater blood flow could deliver more chylomicrons to adipose tissue, potentially increasing the opportunity for additional fat storage and less exaggeration of hypertriglyceridaemia postprandially. Therefore, blood flow to adipose tissue in the postprandial period may regulate regional fatty acid storage. In contrast, in subjects with normal response to OFTT, no gender differences were found in postprandial TG levels. Thus, in subjects with a smaller pool of TG-rich lipoproteins rapid lipolysis might restrict transfer of cholesteryl ester out of HDL surface and limit the effect of high CETP activity linked to the TaqIB polymorphism. This may explain why subjects in the subgroup with normal responses to OFTT, who have very low fasting TG (< 100 mg/dl in both genders) show no gender difference in any of *CETP *polymorphisms. In all groups, hFH women had higher HDL cholesterol levels than men with hFH. This was also reported by others [62].

As already discussed, the TaqI B2 allele has been associated with protection against an exaggerated postprandial TG rise and a subsequent lowering of the HDL cholesterol levels in patients with increased TG concentrations (fasting and/or postprandial) [42]. This was attributed to the B2 lowering-effect on CETP mass and activity which may become more prominent when TG levels are beyond normal [42]. Similarly, restricted CETP activity induced by the B2 allele also may affect the cholesteryl ester content of TG-rich lipoproteins (chylomicrons, VLDLs and their remnants). The lower the CETP activity, the lower the exchange rate of TGs and cholesteryl esters between lipoproteins and the lower the cholesteryl ester content of TG-rich lipoproteins. Consequently, lower cholesterol returns to the liver which may result in higher hepatic expression of LDLR.

A substantial part of our life is spent in the postprandial state. Several authors have described OFTTs to evaluate postprandial lipid metabolism. Postprandial hypertriglyceridaemia is not a uniform abnormality. Its pathophysiology is not yet defined. Possibly, the response to dietary fat is a polygenic phenomenon. There is probably a threshold dietary level that may overwhelm the clearance capacity and thus alters the postprandial lipid composition of circulating lipoproteins. The more the lipolytic pathway is saturated in the fasting state, the greater the magnitude of postprandial triglyceridaemia [46]. The changes in lipoproteins observed postprandially mainly involve a marked increase in TG-rich lipoproteins which promotes the formation of atheromatic small dense LDLs [46]. Additionally, HDLs are transformed into TG-rich HDL particles which become a substrate for hepatic lipase and are cleared more rapidly from the circulation leading to low serum HDL cholesterol levels [46]. As HDL is the key mediator of reverse cholesterol transport [63], low HDL levels may impact this pathway.

From the available data, it is still difficult to establish normal postprandial TG ranges. In Eriksson's et al. study [64] the TG levels were 176 ± 17 mg/dl at 3.5 h. Similar results were found by Boquist et al. [65] (177 mg/dl) and by Eliasson et al. [66] (186 mg/dl). Higher TG levels were found by Weintraub et al. [67] and Cabezas et al. [68], both approximately 212 mg/dl and by Karpe et al. [69] as approximately 248 mg/dl. Our OFTT studies on healthy subjects have revealed a limit range of 220 mg/dl [44], close to the above values, and this was used as a threshold TG level in the current study.

This study has limitations. This is an open cross-sectional study comparing men with postmenopausal women so the effect of menopause on fasting TG levels was not assessed. Hormone evaluation was not performed. However, the classification of women as postmenopausal was based on the absence of menses for at least 12 months [70-72], which is widely accepted. No adjustment for multiple comparisons was applied because every comparison made (for each variable) between genders or allele carriers leads to independent conclusions, rather than representing a whole family of comparisons, where a protection against the inflation of type I error due to multiplicity should be used [73]. Additionally, sub grouping led to small study groups and therefore, a greater number of subjects are needed to draw definite conclusions.

## Conclusion

In conclusion, it seems that gender and TaqIB polymorphism of the *CETP *gene were both associated with the distribution of TG values after OFTT, only in subjects with a pathological response to OFTT. Specifically, men carrying the B2 allele of the TaqIB polymorphism showed a higher postprandial TG peak and a delayed return to basal values compared with women carrying B2. These patients may need a more aggressive hypolipidaemic treatment. In contrast, no gender differences were found postprandially in subjects with normal response to OFTT. Furthermore, there was no influence of the S447X of *LPL *polymorphism on gender. In general, in hFH patients with pathological response to OFTT, the presence of the B2 allele was significantly related to lower levels of TG-AUC, adjusting for gender. However, further investigations in larger populations are required to replicate and confirm these findings.

## Competing interests

This commentary paper was written independently; no company or institution supported it financially. Some of the authors have attended conferences, given lectures and participated in advisory boards or trials sponsored by various pharmaceutical companies.

## Authors' contributions

KKA participated in the development of hypothesis, drafting of the manuscript and carried out the genetic analysis. GDK conceived the study and participated in the development of the hypothesis, the study design and drafting of the manuscript. PMK participated in recruitment of subjects, clinical evaluation and drafting of the manuscript. CM performed the statistical analysis and interpretation of data. GH, CM DD participated in the study design and drafting of the manuscript. DPM participated in revising the manuscript critically for important intellectual content. DVC participated in the study design and its coordination. All authors read and approved the final manuscript.
